# CO_2_-Driven Ocean Acidification Alters and Weakens Integrity of the Calcareous Tubes Produced by the Serpulid Tubeworm, *Hydroides elegans*

**DOI:** 10.1371/journal.pone.0042718

**Published:** 2012-08-13

**Authors:** Vera Bin San Chan, Chaoyi Li, Ackley Charles Lane, Yanchun Wang, Xingwen Lu, Kaimin Shih, Tong Zhang, Vengatesen Thiyagarajan

**Affiliations:** 1 The Swire Institute of Marine Science and School of Biological Sciences, The University of Hong Kong, Pokfulam, Hong Kong, Special Administrative Region, People's Republic of China; 2 Department of Civil Engineering, The University of Hong Kong, Pokfulam, Hong Kong, Special Administrative Region, People's Republic of China; University of Connecticut, United States of America

## Abstract

As a consequence of anthropogenic CO_2-_driven ocean acidification (OA), coastal waters are becoming increasingly challenging for calcifiers due to reductions in saturation states of calcium carbonate (CaCO_3_) minerals. The response of calcification rate is one of the most frequently investigated symptoms of OA. However, OA may also result in poor quality calcareous products through impaired calcification processes despite there being no observed change in calcification rate. The mineralogy and ultrastructure of the calcareous products under OA conditions may be altered, resulting in changes to the mechanical properties of calcified structures. Here, the warm water biofouling tubeworm, *Hydroides elegans*, was reared from larva to early juvenile stage at the aragonite saturation state (Ω_A_) for the current *p*CO_2_ level (ambient) and those predicted for the years 2050, 2100 and 2300. Composition, ultrastructure and mechanical strength of the calcareous tubes produced by those early juvenile tubeworms were examined using X-ray diffraction (XRD), Fourier transform infrared spectroscopy (FT-IR), scanning electron microscopy (SEM) and nanoindentation. Juvenile tubes were composed primarily of the highly soluble CaCO_3_ mineral form, aragonite. Tubes produced in seawater with aragonite saturation states near or below one had significantly higher proportions of the crystalline precursor, amorphous calcium carbonate (ACC) and the calcite/aragonite ratio dramatically increased. These alterations in tube mineralogy resulted in a holistic deterioration of the tube hardness and elasticity. Thus, in conditions where Ω_A_ is near or below one, the aragonite-producing juvenile tubeworms may no longer be able to maintain the integrity of their calcification products, and may result in reduced survivorship due to the weakened tube protection.

## Introduction

As a consequence of anthropogenic carbon dioxide (CO_2_) emissions, the atmospheric CO_2_ level has been increasing in an unprecedented rate. One third of this CO_2_ is absorbed by the oceans through the process called “ocean acidification” (OA) [1]. This excess CO_2_ decreases the pH, the carbonate ion (CO_3_^2−^) concentration and the calcium carbonate (CaCO_3_) saturation states (Ω) in seawater [2]. The Arctic and Southern Oceans are expected to experience aragonite undersaturation before the middle of this century [3]. Similarly, estuaries and coastal waters may experience OA-like conditions sooner than the open ocean because of (a) anthropogenic addition of nutrients and eutrophication which consequently increases CO_2_ concentrations and lowers pH [4], [5], and (b) their inherently weak carbonate buffering capacity [6]. Therefore, top priority should be given to the study of biocalcification in benthic invertebrates and the challenges as in these naturally susceptible and variable coastal habitats [7].

The majority of benthic organisms living in coastal waters have complex life cycle, during which the pelagic larva must select a substrate, attach to it and then metamorphose into a benthic adult. This irreversible pelagic-benthic larval transition is an energetically expensive, delicate, and rapid process [8]. In calcifiers, these newly metamorphosed individuals must build a tough protective calcareous structure as soon as possible using a costly and complex biomineralization process [9], [10]. In most larval forms, the formation of calcified structures starts from an unstable amorphous transient precursor of the calcitic or aragonitic forms of calcium carbonate defined by its irregular lattice structure, called amorphous calcium carbonate (ACC) [11], [12]. ACC is subsequently transformed into the more stable forms of CaCO_3_, aragonite and/or calcite [13]–[16]. Since aragonite is 35% more soluble than calcite [17], aragonite-producing organisms are expected to be especially vulnerable to CO_2_-driven OA [18].

The ultrastructure and mechanical properties of calcareous structures produced under OA conditions, especially during the vulnerable early juvenile stage, may be compromised resulting in reduced survival [19]–[23]. There are several plausible mechanisms by which biomineralization under OA conditions could be affected. In order to cope with OA, organisms might differentially allocate energy, favoring physiological homeostasis over the fabrication of calcareous tubes/skeltons/shells [24], [25]. This change in energy allocation may favor the less costly, but more brittle, calcite polymorph of calcium carbonate. Calcite has lower organic content and a lower packing density of calcium and carbonate ions than aragonite [26]. Additionally, calcifiers experiencing OA stress may not have sufficient energy to construct the matrix proteins which are responsible for enhancing mechanical strength [27]. Secreting matrix proteins, such as the phosphate containing proteins known to stabilize ACC, is a vital process in shell production from the very initial stages often begun during the sensitve pelagic larval phase [28]. Finally, changes in ultrastructure and mechanical strength may be due to the preferential dissolution of more soluble polymorph of calcium carbonate, aragonite. Calcification under OA conditions may result in weaker products, e.g. partial loss of aragonite and/or production of disordered crystal morphologies might result in more brittle calcified structures dominated by poorly ordered calcite crystals. Regardless of the source(s) of alterations to calcium carbonate structures, any difference in the ability to produce orderly layered biominerals may also reduce the effectiveness of shells to protect against external attacks by predators like fishes and crabs [29], [30]. The ability to calcify with structural and mechanical integrity is, therefore, an important focal point when evaluating the calcification responses of marine calcifiers to OA [20], [31].

Among calcifying coastal organisms, the serpulid tubeworm (*Hydroides elegans*) is a commercially important fouling species whose calcification appears to be highly altered by OA [32]. Due to their unique larval metamorphosis pattern, rapid calcification immediately after metamorphosis, short generation time (3 to 4 weeks) and ease of culture in the laboratory, this species has long served as a model for larval biology and biofouling research [33]. Here, this species also provided a unique opportunity for us to examine their juvenile tube (shell) composition, structural integrity and mechanical strength as a function of increasing OA stress. This study sought to determine how OA will alter the composition and structure of the juvenile tube, and to what degree those alterations would affect its mechanical properties such as hardness and elasticity. This comparative analysis of calcareous tubes produced in ambient and OA environments by juvenile tubeworms is a very important first step toward understanding the interaction between biomineral production and the environment, which would ultimately, enabled us to better assess and predict the impact of OA on one of this commercially important calcifier.

## Methods

### Study organism

The calcareous tube forming (tubeworm), *Hydroides elegans* (Haswell, 1883), is a major polychaete species in coastal and estuarine biofouling communities [33]. This cosmopolitan warm water species completes its life-cycle in 3 to 4 weeks [34]. Adults of the tubeworm were collected during their peak reproductive season (January–March, 2011;16–17°C, salinity 30–32 ppt, and pH 8.0 to 8.2) from the floating structures in fish farms located at Yung Shue O, Hong Kong (22°27′N, 114°23′W). Animals were acclimatized in the laboratory for 3 to 7 days in an ambient temperature, pH and salinity condition. The female and male gonads were obtained from >30 randomly selected individuals and mixed to produce one, genetically diverse monoculture [35]. The majority of embryos developed into trochophore larvae within 12 h at 21°C, 33 ppt (salinity) and pH 8.1 [34]. This batch of newly hatched larvae was used for the following CO_2_ perturbation experiment.

### Design of the CO_2_ perturbation experiment

Experimental design and CO_2_ perturbation methodology suggested in the Guide for Best Practices in Ocean Acidification Research (http://www.epoca-project.eu) manual were used in this study to examine the effect of high partial pressure of CO_2_ (*p*CO_2_) or low pH (hereafter referred to as “low pH, high *p*CO_2_” or OA treatments) on calcareous tubes built by the juvenile tubeworm [36]. There were three high *p*CO_2_ treatments namely *p*CO_2_∼850 µatm (pH∼7.9 and Ω_A_∼2), *p*CO_2_∼2000 µatm (pH 7.6 or Ω_A_∼1) and *p*CO_2_∼3000 µatm (pH 7.4 or Ω_A_∼0.7). The control treatment had the ambient *p*CO_2_∼500 µatm (pH 8.1 or Ω_A_∼3). The three high *p*CO_2_ treatment levels were chosen to represent the *p*CO_2_ level predicted by the “business-as-usual” emission scenario for the year 2050, 2100 and 2300, respectively [37]. The selected *p*CO_2_ levels are also commonly observed in the natural carbonate system variability found in the subtropical estuarine and coastal waters where *H. elegans* is found [6], [38], [39].

The high *p*CO_2_ levels were achieved by enriching ambient air with pure CO_2_ using dual (air and CO_2_) variable area flow meter/controllers (Cole-Parmer Inc.). The experiments were conducted in an indoor aquarium room; to minimize the potential for acidification of control aquaria due to atmospheric CO_2_ build up in the room, the air was drawn from outside the aquarium room. To describe the carbonate system and the *p*CO_2_ level, the pH was measured daily throughout the experiment using a Mettler-Toledo pH meter (NBS scale, calibrated with Mettler-Toledo buffers at pH4, pH7 and pH 10; every day prior to measurement of seawater pH). Every two days, total alkalinity was measured by potentiometric titration using the Gran Plot method [40], and checked against seawater reference materials (Batch 98, A.G. Dickson, Scripps Institution of Oceanography). The total alkalinity reading obtained from the reference materials (measured one time in every series of titrations) showed an accuracy that deviated 0.13% to 1.62% from the manufacturer's value. All carbonate chemistry parameters for each experimental tank were calculated from the measured values of temperature, pH, salinity and total alkalinity using the CO2SYS program [41] using the values for carbonic acid dissociation constants K_1_* and K_2_*of Millero [42].

The newly hatched trochophore larvae (∼12 h post-fertilization) were divided into 20 groups and randomly assigned into treatment and control culture tanks. There were 20 tanks in total (4 treatments including control ×5 biological replicate tanks; each tank provides an independent sample of multiple tubeworms for analyses). Each tank received ∼3500 larvae (2 larvae ml^−1^). Larvae were reared in plastic tanks containing 1.7 L of 0.22 µm filtered seawater using optimal food concentration (1.8×10^5^ cells mL^−1^of *Isochrysis galbana*; in 10 folds greater than the optimum concentration of 1.8×10^4^ cells mL^−1^ [43], this ensure unlimited development of the tubeworm larvae by food supply), temperature (25°C) and salinity (33–34 ppt) [34], culture water was renewed every other day. Once larvae reached competency to settle (attach) and metamorphose into juvenile tubeworms, i.e. 5 days, they were induced to attach on plastic surface using an artificial inducer of attachment, 10^−4^ M IBMX [33]. This well-known inducer was used instead of natural inducers such as biofilms in order to synchronize larval attachment and metamorphosis as well as to trigger the calcification immediately after metamorphosis especially at the lowest pH treatment levels where natural calcification is delayed [32]. Larvae that successfully attached and metamorphosed with calcareous tubes were kept in the respective treatment and culture conditions for 9 days, after which the calcareous tubes were collected for tube structure, composition and mechanical property analyses.

### Preparation of samples for tube analyses

Fine powder, fractures and cross-sections of tube samples were prepared for structural analysis using X-ray powder diffraction (XRD), Fourier transform infrared spectrometry (FT-IR), scanning electron microscopy (SEM) and nanoindentation. Individuals were first rinsed with double-distilled water to remove salts, dislodged from the substrate and then preserved in 75% ethanol. Soft organic tissues were removed by immersing the tubeworms in 5% bleach (NaOCl, Clorox™) for about 20 minutes [44]. These tissue free tubes were processed further into fine and homogenous powder for XRD and FT-IR analyses. Grinding was performed in a 75% ethanol medium to minimize structural damage caused by over-grinding [45], glass microscope slides were used to provide a non-adhesive surface on which the slurry samples were air-dried overnight. Weighing papers sachets were used to store the powder samples (1–18 mg, at 20–24°C) until analysis.

Tube fractures and sections were prepared for ultrastructure analysis using scanning electron microscope (SEM). To create fractured surfaces, tissue free tube samples were frozen for 30 seconds in liquid nitrogen, and then immediately crushed using fine forceps to reduce grinding action during fracture. To prepare sections, tubes were embedded in resin, and then sectioned perpendicular to the long axis of the tube exposing the cross-sectional surfaces using a handmade low speed saw in the hard tissue laboratory of the Prince Philip Dental Hospital, Hong Kong. After locating the cross-sectional surface, excess surrounding resins were trimmed away to produce a small sectioned area suitable for ultramicrotomy [46]. Smooth, polished surfaces were obtained using an ultramicrotome to remove thin (85 nm thick) sections (Ultracut S, Leica), and the surface was etched in 1% acetic acid solution for 10 second to reveal topographical details of the microstructures prior to SEM.

### Analysis of tube composition

Relative proportion of calcite, aragonite, and amorphous CaCO_3_ (ACC) content in tube samples was qualitatively and semi-quantitatively analyzed using XRD and FT-IR. In the XRD samples, a known quantity of calcium fluoride (CaF_2_) was mixed with powder tube samples as an internal standard [47]. XRD spectrum and data were obtained using a Bruker D8 Advance X-ray powder diffractometer equipped with a Cu Kα radiation and a LynxEye detector. The system was calibrated by Standard Reference Material 660a (lanthanum hexa boride, LaB_6_), obtained from the U. S. National Institute of Standard and Technology, for the line position. The diffractometer was operated at 40 kV and 40 mA, and the 2*θ* scan range was from 10° to 110°, with a step size of 0.02° and a scan speed of 0.3 s/step. Qualitative phase identification was performed using Eva XRD Pattern Processing software (Bruker Co. Ltd.) by matching powder XRD patterns with those retrieved from the standard powder diffraction database of the International Centre for Diffraction Data (ICDD PDF-2 Release 2008). The Rietveld refinement method for quantitative analysis of the phase compositions was processed by the TOPAS (version 4.0) crystallographic program [48].The elemental proxy of calcite mineral in tube samples, the magnesium/calcium ratio, was also measured using the housed EDS system in SEM in order to corroborate the results of XRD.

ACC content was quantified using the intensity ratio of carbonate ion infrared absorption bands, I_max_ *ν*_2_/I_max_ *ν*_4_, using FT-IR [16], [49]. To obtain infrared absorption spectra, powdered samples (∼1 mg) were mixed with KBr (∼10 mg; dehydrated at 98°C overnight). This mixture was pressed into a 13 mm diameter pellet (at 9 tons; for 2 min). Fourier transform infrared spectrometer (FT-IR, L120-000B, Perkin Elmer, USA) was used to obtain the spectrum ranging from 500–2000 cm^−1^ with 1 cm^−1^resolution. The spectra were baseline corrected using Origin Pro Version 8.5. Second order derivatives were obtained by the Savitzky-Golay smoothing method before the peak heights at 855 cm^−1^ (*ν*_2_) and 713 cm^−1^ (*ν*_4_) corresponding to the internal vibration modes of CO_3_^2−^ ions in aragonite were measured. The ratio of the maximal intensities of the *ν*_2_ versus the *ν*_4_ absorption bands I_max_ *ν*_2_/I_max_ *ν*_4_ was quantified to determine the effect of pH/*p*CO_2_ on ACC content in tubes.

Tube ultrastructure was examined in fractured and cross-sectioned samples using SEM. Tube sections and fractures were mounted onto aluminum stubs with carbon tape and were sputter coated with gold – palladium alloy (∼50 nm) prior to SEM study. Specimens with poorly conductive resin were surrounded with silver paint to avoid electron charging. SEM analysis was performed using Leo 1530 FEG SEM, equipped with an Inca EDX system [50]. To validate the observed difference in calcite content, Mg/Ca ratio, also the known elemental proxy of calcite was calculated from the ratio of elemental Mg and Ca composition obtained from EDX measurement (at 20 kV) from 15 random points on the fractured surfaces [51].

### Tube mechanical strength

Tube mechanical properties, hardness and elasticity, were measured using nanoindentation. After SEM imaging, resin embedded specimens were further cut with a diamond knife to remove thin (85 nm thick) sections with an ultramicrotome (Ultracut S, Leica). This removed the gold-palladium alloy coating use in the SEM analysis, and also smoothed the surface topography for nanoindentation testing. Specimens were tested dry in ambient conditions using a triboindenter (Hysitron Inc., USA) equipped with in-situ scanning probe microscopy (SPM) imaging for topographic imaging of residual impressions. For each specimen, measurements were taken from at least six random locations of the tube section. Attention was paid to avoid measurements from the bottom adhering structures of the tube, which can have distinctive mineralogy and architectures as shown in other calcifiers like barnacles [52]. Dynamic load and displacement of the quartz Berkovich three-sided pyramid indenter was monitored at a load resolution of <1 nN and displacement resolution of 0.1 nm. Specimens with little surface roughness were indented with controlled peak loads between 500 to 7000 µN, in order to achieve necessary depth of penetration from 130 to 700 nm that minimizes the error in determining of contact area between the indenter and the specimen [53]. The applied load function was divided into three segments: the first segment was a loading phase with a standard loading time of five seconds, the second segment was a hold period of three seconds, and the third segment was an unloading phase with decreasing load at the same rate as first segment until zero force was reached. Hardness (H) and elastic modulus (E) generated from the recorded load-displacement curves were recorded after each measurement [54].

### Data analysis

The effect of elevated *p*CO_2_ on the tube composition and mechanical properties were assessed using one factor analysis of variance (ANOVA). Before the analysis, data were checked for ANOVA assumptions such as normality and heterogeneous expectations using Shapiro–Wilk's and Fligner-Killeen tests, respectively. If the ANOVA test showed a significant difference among treatments, the Dunnett test was used to compare the effect of each *p*CO_2_ treatment compared with the control. Carbonate system parameters were analyzed for differences among treatments using one-way ANOVAs and post-hoc Tukey HSD tests.

## Results

### CO_2_ perturbation and carbonate chemistry

**Figure 1:**
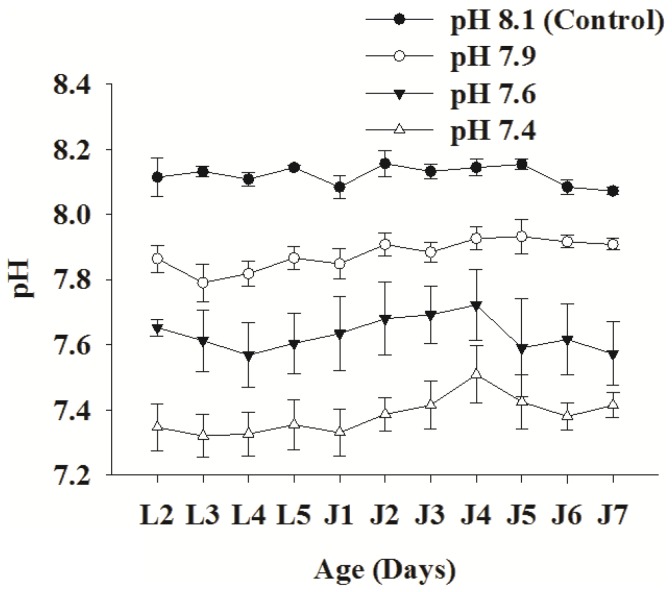
Variation of pH (NBS scale), a proxy of carbonate system, in control and among pH/*p*CO_2_ treatment culture tanks during the experiment. Each data point represents mean ± SD of 5 biologically independent measurements, pH levels are statistically different with p-value<0.05. Abbreviations: L1–L5, Day 1–5 of larval phase; J1–J7, Day 1–7 of juvenile phase.

The measured and calculated carbonate system parameters during the study period are shown in Table 1. As expected, there was a significant difference in pH, *p*CO_2_ and aragonite saturation (Ω_A_) levels between the control and the given treatment conditions (ANOVA: pH, F_3, 16_ = 61.445, p<0.001; Ω_A_, F_3, 16_ = 96.971, p<0.001). The Ω_A_ in the culture tanks of the pH 7.6 and 7.4 treatment groups were near and below saturation levels, respectively. However, there was no significant difference in salinity, total alkalinity and temperature among control and treatments (one-way ANOVA: Salinity; F_3, 16_ = 1.071, p>0.05; total alkalinity, F_3, 16_ = 4.482, 0.01<p<0.05; temperature, F_3, 16_ = 2.339, p>0.05; Tukey post hoc test; p>0.05 for all comparisons of experimental groups). The daily pH measurements and subsequent calculation of the carbonate chemistry parameters indicated that the carbonate system within and among treatment tanks was relatively stable throughout the experiment (Figure 1). The number of algal cells used to feed larvae or juveniles did not significantly affect aragonite saturation level (Figure S1). Such a stable carbonate system via experimental CO_2_ perturbation allowed us to examine the effects of high *p*CO_2_ on tube composition, ultrastructure and mechanics.

**Table 1:** Measured and calculated values (mean ± SD, n = 5) of environmental and carbonate system parameters for the ambient (control) and the three pH/*p*CO_2_ treatments before sample collection for shell structure, composition and mechanical strength measurement at two monitoring time points, day 5 juvenile/J5 and day 7 juvenile/J7.

	Measured parameters				Calculated parameters	
CO_2_ Treatment	pH	Salinity ppt	Temp °C	TA (µmol/kg-SW)	*p*CO_2_ (µatm)	Ω_A_
Day 5						
Control	8.05±0.03	34.8±1.2	25.6±0.1	2160±71	539±42	2.5±0.3
Low	7.88±0.04	34.5±1.1	25.6±0.1	2293±97	874±88	1.8±0.2
Medium	7.67±0.04	35.3±0.8	25.6±0.1	2262±48	1530±142	1.2±0.1
High	7.34±0.08	34.5±1.1	25.6±0.1	2326±70	3548±675	0.6±0.1
Day 7						
Control	8.08±0.02	34.4±0.5	25.6±0.2	2210±112	514±27	2.7±0.2
Low	7.89±0.03	34.5±0.6	25.1±0.5	2212±60	852±75	1.8±0.1
Medium	7.58±0.11	34.2±0.8	25.4±0.3	2346±106	2041±592	1.1±0.2
High	7.37±0.14	33.7±1.0	25.6±0.3	2356±41	3438±982	0.7±0.2

TA, total alkalinity of seawater; pCO2, partial pressure of CO2; ΩA, aragonite saturation state.

### Tube composition

**Figure 2:**
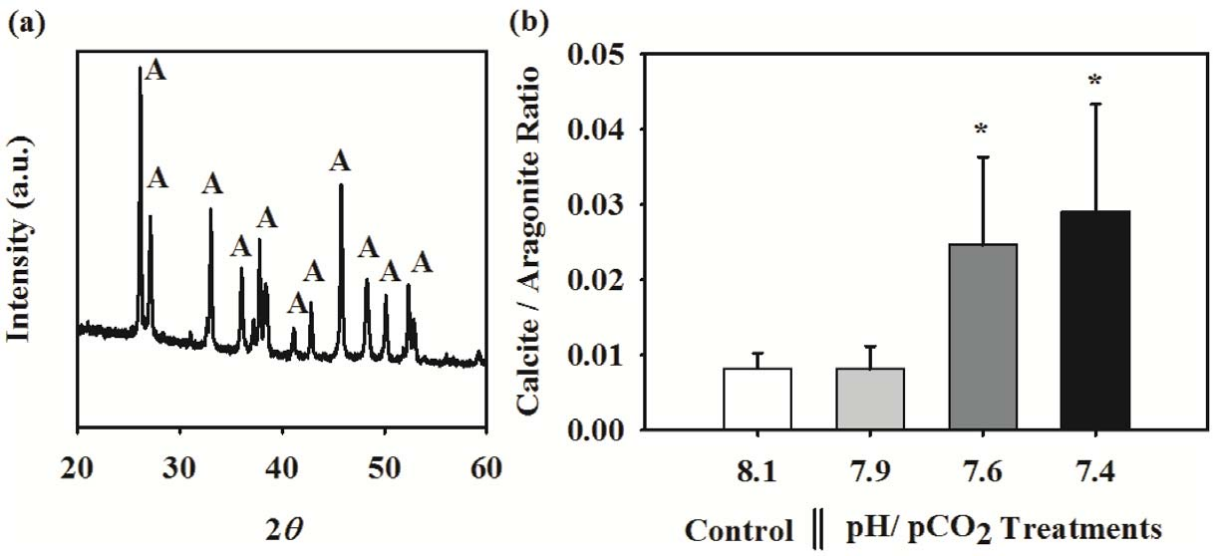
Effects of pH/*p*CO_2_ on shell mineralogy in the juvenile tubeworm, *Hydroides elegans*. (a) The X-ray diffraction pattern of shell obtained from the control pH/*p*CO_2_ (pH 8.1) showing the presence a set of diffraction peaks resulting from the presence of the dominating aragonite (A) phase. (b) Effect of pH/*p*CO_2_ on the shell calcite/aragonite ratio, which was quantified from the X-ray diffraction analysis. Each bar represents the mean ± SD of 5 biologically independent measurements each obtained from independent culture. The asterisk (*) shown on the bar indicates a significant difference between the control and the treatment according to the Dunnett's Post-hoc test (p<0.05).

**Figure 3:**
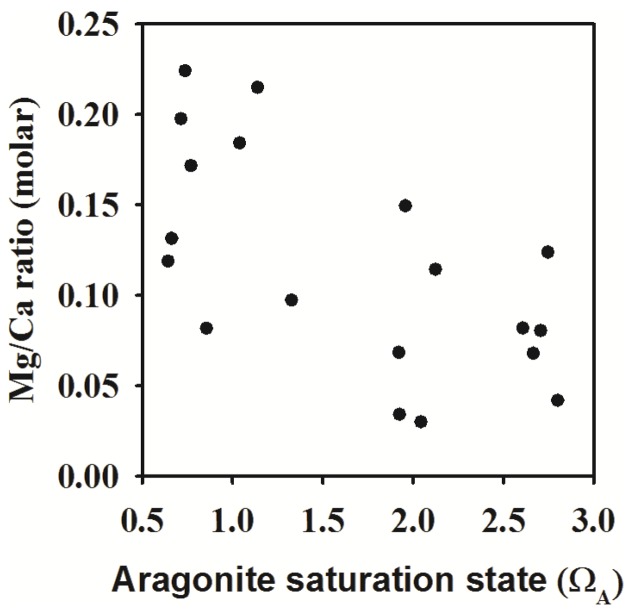
The relationship between aragonite saturation state and the tube magnesium to calcium (Mg/Ca) ratio. The strength of the relationship was analyzed using the Spearman's rank-order correlation coefficient.

The XRD diffraction peaks corresponding to the tube mineral indicated the presence of mixture phases of aragonite and structure of low-Mg calcite. The calcite main peak *2θ* position at 29.4 degree is consistent with a calcite structure with low Mg occupancy. In ambient seawater chemistry conditions (control), the juvenile tube was predominantly composed of aragonite with negligible calcite content (Figure 2a), with a calcite/aragonite ratio of ∼0.008. Surprisingly, this ratio increased by 2 to 3 times at elevated *p*CO_2_ levels, i.e. pH 7.6 and 7.4 (Figure 2b; ANOVA results: F _3, 16_ = 6.77, p<0.05). However, the relative proportion of aragonite and calcite in the tube was not affected by the near future *p*CO_2_ level of pH 7.9. This increase in calcite content at elevated *p*CO_2_ conditions was also confirmed using the energy-dispersive X-ray spectroscopy (EDS or EDX) analysis of magnesium to calcium ratio. The Mg/Ca ratio in tube, an elemental indicator for calcite, also increased with decreasing pH or aragonite saturation level (Figure 3; Spearman Rank Correlation Test result: R_spearman_ = −0.58, p<0.05).

**Figure 4:**
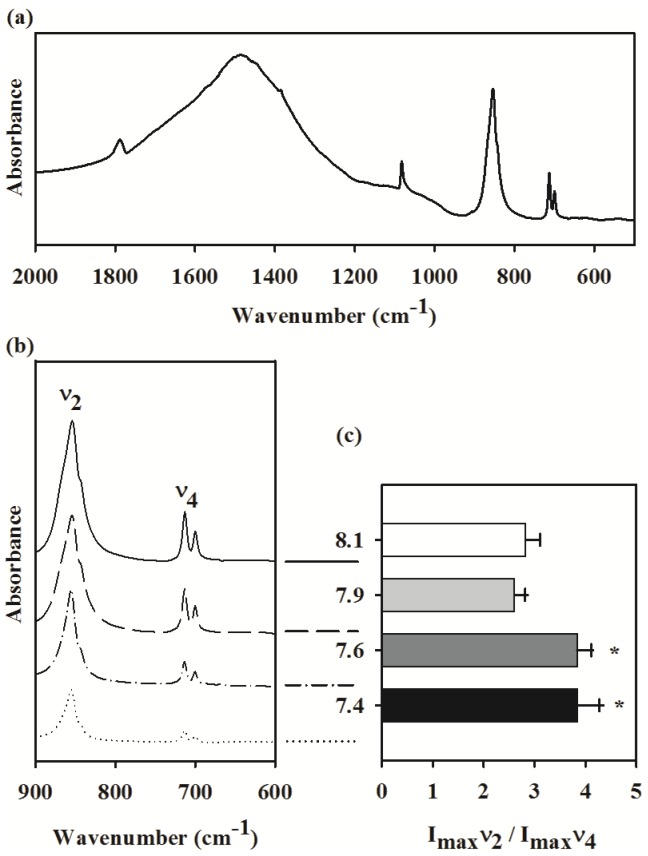
Effects of pH/*p*CO_2_ on shell mineralogy in the juvenile tubeworm, *Hydroides elegans*. (a) The infrared absorption spectrum obtained from shell samples of the control pH/*p*CO_2_ (pH 8.1) using the Fourier transform infrared spectrometry (FT-IR). (b) Effects of pH/*p*CO_2_ on the magnified portion, between wavelengths 600–900 cm^−1^, of infrared spectral pattern of the shell. (c) Effect of pH/*p*CO_2_ on the intensity ratio (I_max_) of the two absorption peaks at 855 cm^−1^ (*ν*_2_) and 713 cm^−1^ (*ν*_4_). The I_max_ *ν*_2_/I_max_ *ν*_4_ ratio is an indicator of amorphous calcium carbonate (ACC) content. Each bar represents the mean ± SD of 5 biologically independent measurements. The asterisk (*) shown on the bar indicates a significant difference between the control and the treatment according to the Dunnett's Post-hoc test (p<0.05).

The FT-IR spectra of the tube built by the tubeworm at ambient seawater condition is shown in Figure 4a.The IR peaks at 855 cm^−1^ and 713 cm^−1^ were assigned as ν_2_ and ν_4_ absorption bands of the carbonate groups of the crystalline CaCO_3_, respectively [16]. The intensity ratio of ν_2_ to ν_4_ was used to quantify the amorphous CaCO_3_ content (ACC) in the tube (Figure 4b). The ratio ranged between 2.5 to 3 for the tubes obtained from the ambient condition. The high *p*CO_2_ treatments significantly increased the ACC content in the tube (Figure 4c; ANOVA results: *F*_3, 14_ = 20.01, *p*<0.001). Although the tube ACC content at the pH 7.9 was statistically similar to the ambient pH 8.1, the two low pH treatments 7.6 and 7.4 had significantly higher amount of ACC (Figure 4c).

### Tube ultrastructure

**Figure 5:**
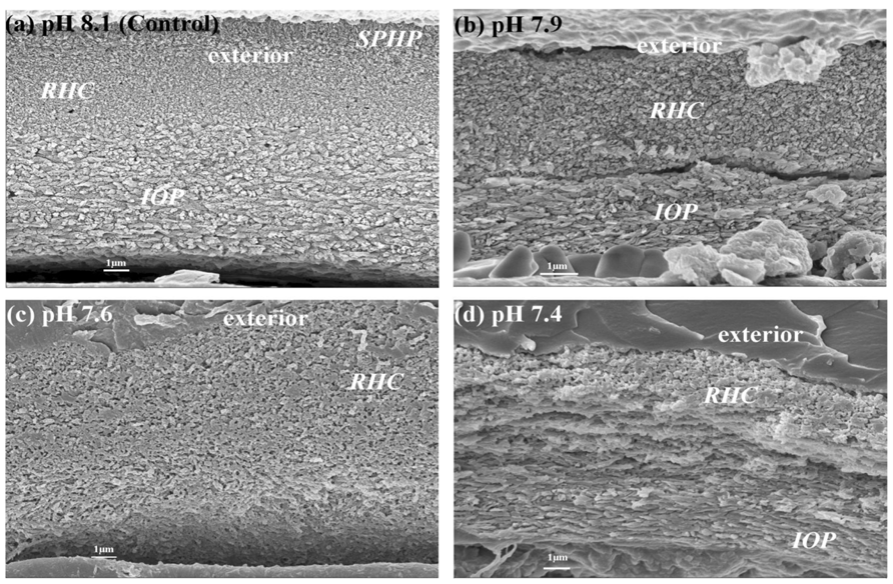
Effects of pH/*p*CO_2_ on cross sectional views of the tube ultrastructures in the juvenile tubeworm, *Hydroides elegans*. Abbreviations: SPHP, spherulitic prismatic structure; IOP, irregularly oriented prismatic structure; RHC round homogenous crystal structure.

**Figure 6:**
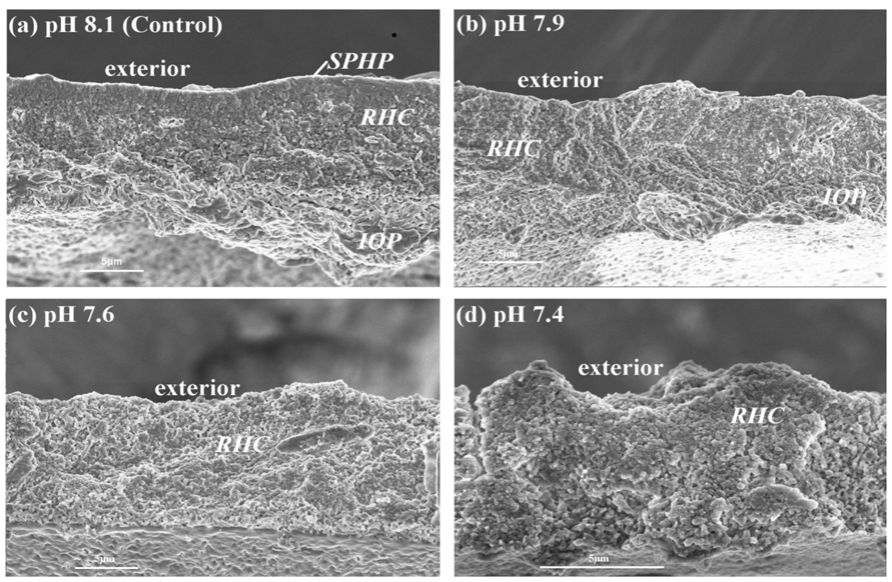
Effects of *p*CO_2_ on fracture surfaces of the tube ultrastructures in the juvenile tubeworms, *Hydroides elegans*. Abbreviations: SPHP, spherulitic prismatic structure; IOP, irregularly oriented prismatic structure; RHC round homogenous crystal structure.

The scanning electron micrograph (SEM) of tubeworm tube ultrastructure is shown in Figures 5 and 6. Using the recently established terminology and description for the tubeworm tube ultrastructure analysis [55], three distinguishable layers were observed in the juvenile tubeworm tube raised at ambient pH 8.1. The tube is coated by the outermost layer of spherulitic prismatic structure (SPHP), the middle layer composed of rounded homogeneous crystal structure (RHC), and the innermost layer is made of an irregularly oriented prismatic structure (IOP).Unlike adult tubes, the juvenile tubes examined in this study did not have a prominent SPHP layer (Figure 5a). Tube sections as well as fractures had all the three layers (Figures 5a and 6a). The high *p*CO_2_ had a significant impact on the tube ultrastructure. For example, tubes produced at the pH treatments 7.6 and 7.4 had higher porosity and layer irregularity with more signs of pitting (Figures 5c and d; Figures 6c and d). At all the three high *p*CO_2_ treatments, the IOP layer contained less structured crystallites and thus this layer became indistinguishable from the rounded crystallites observed in RHC layers (Figures 5c and 6d). Under low pH conditions, the crystallites within RHC layer appeared as amorphous cryptocrystalline masses (Figure 5d and 6d).

### Tube mechanical properties

**Figure 7:**
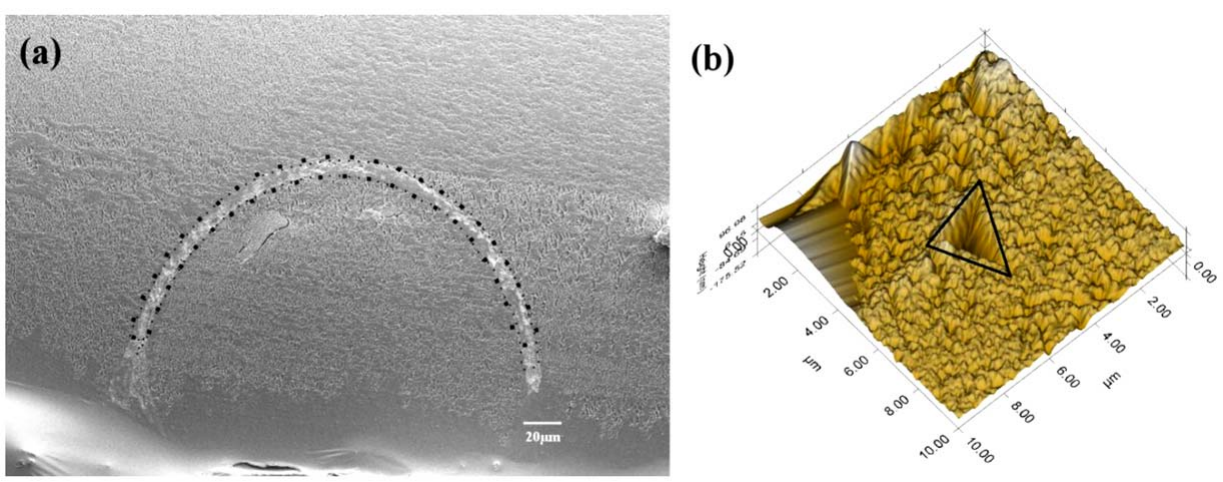
The tube portion used for nanoindentation analysis. (a) Portions of cross-sectional surfaces subjected to of nanoindentation test, at least six random points were tested over the tube area (surrounded by dotted line). (b) A mark created after indentation test from a shell sample of the control pH/*p*CO_2_ (pH 8.1).

**Figure 8:**
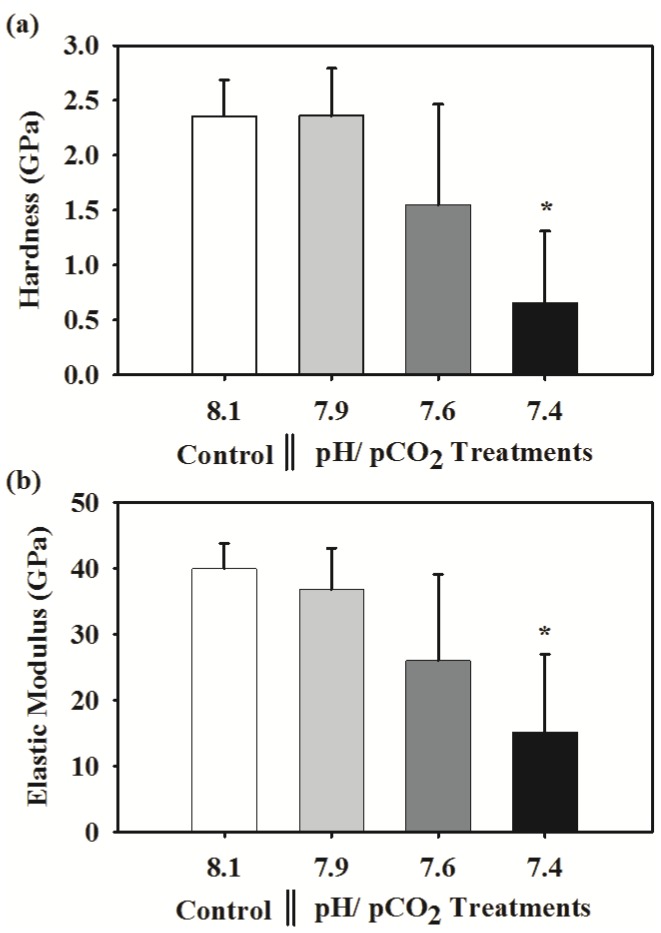
Effects of pH/*p*CO_2_ on the shell hardness and elastic modulus in the juvenile tubeworm, *Hydroides elegans*. Each bar represents the mean ± SD of 4 to 2 biologically independent measurements; i.e. n = 4 for control, n = 3 for the pH 7.9 and 7.6, and n = 2 for the pH 7.4. This uneven biological replicates across treatments was due to loss of samples during hardness measurement. However, the Dunnett test is robust and suitable for the comparison of the mean obtained from uneven sample size. The asterisk (*) shown on the bar indicates a significant difference between the control and the treatment according to the Dunnett's Post-hoc test (p<0.05).

The tube portions where hardness and elasticity measurements were made at different position along the cross sectional surface of the tube as shown in Figure 7a and the typical mark created by the indentation is shown in Figure 7b. At ambient conditions, the tube hardness (H) was 2.36±0.27 GPa and the stiffness (*E*) was 39.94±3.85 GPa. These two mechanical properties of a tube were significantly affected by high *p*CO_2_. At pH 7.4, the hardness and the stiffness were reduced by as much as 72% (F_3,8_ = 4.688, p<0.05) and 62% (F_3,8_ = 4.421, p<0.05), respectively (Figures 8a and b).

## Discussion

In highly productive coastal and estuarine waters, an anthropogenically imposed or naturally decreasing carbonate ion supply due to ocean acidification and/or eutrophication (including hypoxia and upwelling) may hinder or disrupt the calcification of a variety of marine organisms possibly by making this key physiological process more expensive [24], [56]–[58]. Early life stages such as larvae and newly metamorphosed juvenile stages are particularly vulnerable to this unprecedented OA stress due to their high dependence on their environment to provide the appropriate conditions [59]–[65]. Few recent studies on calcifying animals have made pioneering observations on crystallography of calcified structures produced at high *p*CO_2_, including measurements of the elemental calcite/aragonite ratio and microhardness of skeletons [19], [20], [22], [66]. According to these studies, OA has damaging impacts not only to calcification rate but also to the microstructures and crystallographic textures of calcareous products. Organisms are thus faced with twin OA threats, i.e. decreased calcification and abnormal tube microstructure. It is therefore likely that the intensively calcifying juvenile tubeworms will not have the ability to produce a calcareous protective tube with normal structural integrity and mechanical properties when exposed to OA conditions projected for the year 2100 and beyond. This study tested this hypothesis by simultaneously measuring mineral composition, ultrastructures and mechanical properties of CaCO_3_ tubes produced by tubeworms at various OA scenarios finding that high *p*CO_2_ treatments altered tube composition, structure, and hardness. This study also provides a comprehensive analysis of tube mineralogy produced by early juveniles of the important biofouling tubeworm, *Hydroides elegans*.

### Tube mineralogy: Influence of OA

At ambient pH, the tube is primarily made of the more soluble CaCO_3_ polymorph, aragonite, appearing as needle-like prismatic crystallites; and had only a trace amount of the most stable crystalline form of CaCO_3_, calcite. There was also a small but detectable amount of an unstable amorphous form of CaCO_3_, ACC. In contrast, tubes produced by adult tubeworms, *H. dianthus*, *H. norvegicus* or *H. spongicola* had both aragonite and calcite in comparable quantities and thus produced bimineralic calcareous tubes [20], [67], [68]. In tubeworms, there appeared to be an ontogenetic shift from preferentially calcifying aragonite from ACC in the early juvenile, and then both calcite and aragonite from the ACC precursor in adults (Vinn, personal communication). However, this hypothesis is yet to be confirmed in serpulid worms, although the production of calcite or aragonite from the ACC precursor is very common in marine organisms such as molluscs and echinoderms [12], [15].

The most unexpected finding was that the composition of the aragonite dominated bimineralic calcareous tube produced by the juvenile tubeworm is strongly dependent on Ω_A_. In the highest two *p*CO_2_ treatments, when Ω_A_ was near or below one, the tube had significantly higher proportion of calcite (Figure 2b). The EDX-SEM analysis of Mg/Ca ratio (Figure 3) reconfirmed that calcite content increased in response to decreasing Ω_A_. The aragonite to calcite ratio appears to be a phenotypically plastic trait in tubeworms changing with the seawater chemistry. In corroboration with our results, a whelk and another species of a tubeworm had higher calcite/aragonite ratio in Ω_A_ under-saturated conditions [20], [69]. These results can be interpreted as a consequence of preferential dissolution of the more soluble aragonite [20]. Aragonite generally tends to dissolve at a much faster rate near or below Ω_A_ [30]. Juvenile worms of the *H. elegans* are under particularly high risk at low Ω_A_ because (1) calcareous tubes are produced from glands located under the unfolded collars, a calcification site that is semi-exposed to the external environment [70] and (2) the outer surface of the tube structure is protected only by a relatively thin organic layer when compared to bivalves, urchins and corals [20].

In addition to the direct dissolution threat, aragonite production also has a greater energetic requirement than calcite [71]. Accreting aragonite is more costly because it has higher packing density of 2.95 gcm^−3^, while that of calcite is only 2.72 gcm^−3^ [26]. Additionally, aragonite is constructed with expensive organic matrix proteins often in larger quantity than calcite [72]. Since the basic metabolic demands for cellular maintenance, such as ion regulation and protein synthesis, may be increased at low pH [66], [73], budgeting enough energy for aragonite accretion may not be possible. Although the degree of plasticity in polymorph production of these bimineralic calcifiers is still uncertain, calcifying more calcite may be a preferable strategy to enable more economical calcification and better resistance to dissolution in low pH environments. However, the preferential use of calcite as a defense against dissolution should be, at this stage, advanced with caution; because the solubility of this biomineral is also greatly dependent on the crystal sizes and the quantity and nature of organic matrices.

The FT-IR analysis showed an interesting phenomenon, in that the ACC content in the tube increased by 20 to 30% at near or below aragonite saturation. While no certain explanation for this relationship between ACC content and decreasing Ω_A_ can be established by this work, ACC is a known precursor to both aragonite and calcite [49] and increased ACC content at low pH could reflect an increased calcification effort in this tubeworm. Increasing the ACC content may be an attempt to elevate production of crystalline products, i.e. calcite and/or aragonite. Our result showed a change in composition of tubes at pH 7.4, while the animals at pH 7.9 and 7.6 were comparable to the ambient control conditions of pH 8.1. This similarity in treatment effects, however, should be comprehended with caution because of low replication numbers limiting statistical power.

Greater energetic costs in stressful environments, often interpreted as compensatory efforts against corrosive OA environments, are observed as changes in respiration and metabolic activity (in copepods) [74]. It has also been shown that nacre surface can be maintained in corrosive calcification fluid when food levels were increased [75]. The observed changes in skeleton composition such as Mg/Ca ratio can also be a result of altered physiology, which alters the materials for building up mineral products. When Mg/Ca levels were measured in similar OA studies, a reduction of Mg/Ca was found in a coral and echinoderms [76], [77], while Mg/Ca was independent from *p*CO_2_ levels in foraminifera, both findings contrast with the increase in Mg/Ca ratio observed in *H. elegans*. These discrepancies can be a result of variations in calcification pathways, since the fractionation of Mg/Ca in skeletons is determined by the mechanism of biomineralization [78]. The tubeworm in this study produces both calcite and aragonite, so the dissolution of aragonite which causes more calcite component to remain in the skeletal structure, can lead to a higher Mg/Ca as calcite generally incorporates more Mg in the lattice structure [79].

Mg/Ca ratio is recognized to correlate with certain environmental conditions, specifically with water temperature [80], [81], and sometimes salinity [82]. Both temperature and salinity have been maintained constant in the course of this study, so it is reasonable to expect that environmental pH may influence the Mg/Ca ratio in the shell. Cellular acid-base balance and energy metabolism are known to be sensitive to pH alteration in the environment [83], these altered physiological processes may in turn affect shell morphology and composition including Mg/Ca ratio, for example known in foraminifera [84]. This interaction between seawater carbonate chemistry and the ultimate shell features, and how they interact with, or are affected by, the animal's physiology and intracellular environment should be explored further in marine organisms.

### Tube ultrastructure: Influence of OA

The calcareous tube produced by the juvenile tubeworm consists of three distinct textural layers: the outer, middle and inner layers namely spherulitic prismatic structure (SPHP), rounded homogeneous crystal structure (RHC) and irregularly oriented prismatic structure (IOP) respectively. The unoriented RHC and IOP layers usually have low density and weak mechanical resistance. In contrast, the outer most, oriented, SPHP layer is made of densely and orderly packed crystallites effectively protecting the underlying fragile tube structures from dissolution and external attacks [85]. A tube built without thick SPHP is also prone to the propagation of cracks [67].

The OA treatments dramatically affected the orientation, orderly structure and thickness of each of these three mineralized layers (Figures 5 and 6). Although the precise quantitative relationship between OA and tube ultrastructure (e.g. layer thickness and porosity) was not determined in this study, tubes made at all the three low pH treatments showed clear signs of dissolution of the protective SPHP layer, and also appeared to have increased pitting and a more open packing. Both the inner unoriented layers, RHC and IOP, showed a noticeably degraded crystalline structure under OA conditions. The RHC layer appeared corroded with pitting and less densely packed crystals at the lowest pH (Figures 5d & 6d). A similar change in crystallite morphology in response to reduced Ω_A_ in the calcification fluid, influenced by the external Ω_A_, was observed in oysters [20], [86], corals [19], bivalves [87] and the pearl oyster [31]. Under lower internal Ω_A_, nucleation and growth of CaCO_3_ crystallites was negatively affected in corals and resulted in shorter, wider and more faceted crystallites [19]. It may be the case in our observations that the semi-exposed nature of the calcification site resulted in reduced aragonite saturation, resulting in pitting and poorly ordered crystallites as in the aforementioned studies.

### Tube mechanical properties: Influence of OA

Tube hardness and elasticity of the biofouling tubeworm, *Hydroides elegans*, in response to OA was impaired, with reductions in hardness and elasticity of 72% and 62%, respectively. These changes likely result from the disorganized ultrastructure and higher proportion of calcite (or less aragonite) in tubes produced in Ω_A_ undersaturated condition (pH 7.4) [26]. Similar reductions in mechanical properties were found in juvenile oysters [22], [66], the California mussel, *Mytilus californianus* [88], and the pearl oyster [31]. Similar dissolution of aragonite has also been found in Antarctic bivalves [87], such a change in aragonite crystallography and mineralogy also strongly affects the material's hardness and mechanical properties [89].

The observed reduction in tube hardness and elastic modulus can be attributed to either 1) reduced aragonite content of the tube (i.e. reduced calcite/aragonite ratio) or 2) concurrent increase in elemental Mg/Ca ratio) or 3) impaired or altered tube ultrastructure (or increased tube porosity). Aragonite rich calcareous structures are found to have a higher hardness and elasticity than that of calcite dominated crystal structures [90], [91]. In addition, layered structures are generally harder [92]–[94]. Therefore it is reasonable to speculate that *H. elegans* tubes will produce weaker and less elastic tubes under projected OA conditions due to decreased aragonite content and inability to produce multi-layered calcareous structures. According to recent studies, hardness of calcareous structures also depends on organic matrix proteins occluded in the structure [95]. Therefore, OA may have also reduced hardness through various mechanisms, yet results of the present study are insufficient to isolate the cause of such reduction in shell strength.

Although the organic matrix has been reported to show little response to OA in bivalves [31], [96], this ability may be species specific. Both the quality and quantity of organic contents play a crucial role in governing shell formation, and the overall mechanical properties [97]. Thus near or below saturation levels of seawater could pose an important mechanical challenge to tubeworm juveniles. As a consequence of this poorly assembled and fragile tube, juvenile tubeworms may not be able to withstand physical disturbances and predation in the future ocean. In the face of these unprecedented threats, future studies should investigate this biomineral-environment interaction in long-term CO_2_ perturbation experiments involving juveniles as well as adults with special attention to the potential ecological consequences of the altered mechanical properties. Ultimately, a tubeworm's ability to maintain a tube with the appropriate compositions of the particular polymorphs and elemental ratio for the necessary mechanical properties may be affected by low pH, and might subsequently determine their performance as *p*CO_2_ increases.

## Supporting Information

Figure S1 Effects of algae concentration on aragonite saturation state after 24 h and 48 h. This additional experiment without larvae examined the influence of three algal concentrations (0 cell/mL, 10^4^ cells/mL, 10^5^ cells/mL) on the aragonite saturation at four levels of pH (8.1, 7.9, 7.6, 7.4). Each bar represents the mean ± SD of 3 replicates. Three-way ANOVA: Algae; F_2, 48_ = 1.607; p>0.05; Algae*CO_2_; F_6,48_ = 1.444; p<0.05; Algae*Day; F_3,48_ = 0.183; p>0.05; Algae*CO_2_*Day; F_6,48_ = 0.640.
(TIF)
