# Supplementary figures and images for: CO2-Driven Ocean Acidification Alters and Weakens Integrity of the Calcareous Tubes Produced by the Serpulid Tubeworm, Hydroides elegans
Source: PLoS One. 2012 Aug 13;7(8):e42718. doi: 10.1371/journal.pone.0042718 (PMC3418283; doi:10.1371/journal.pone.0042718)

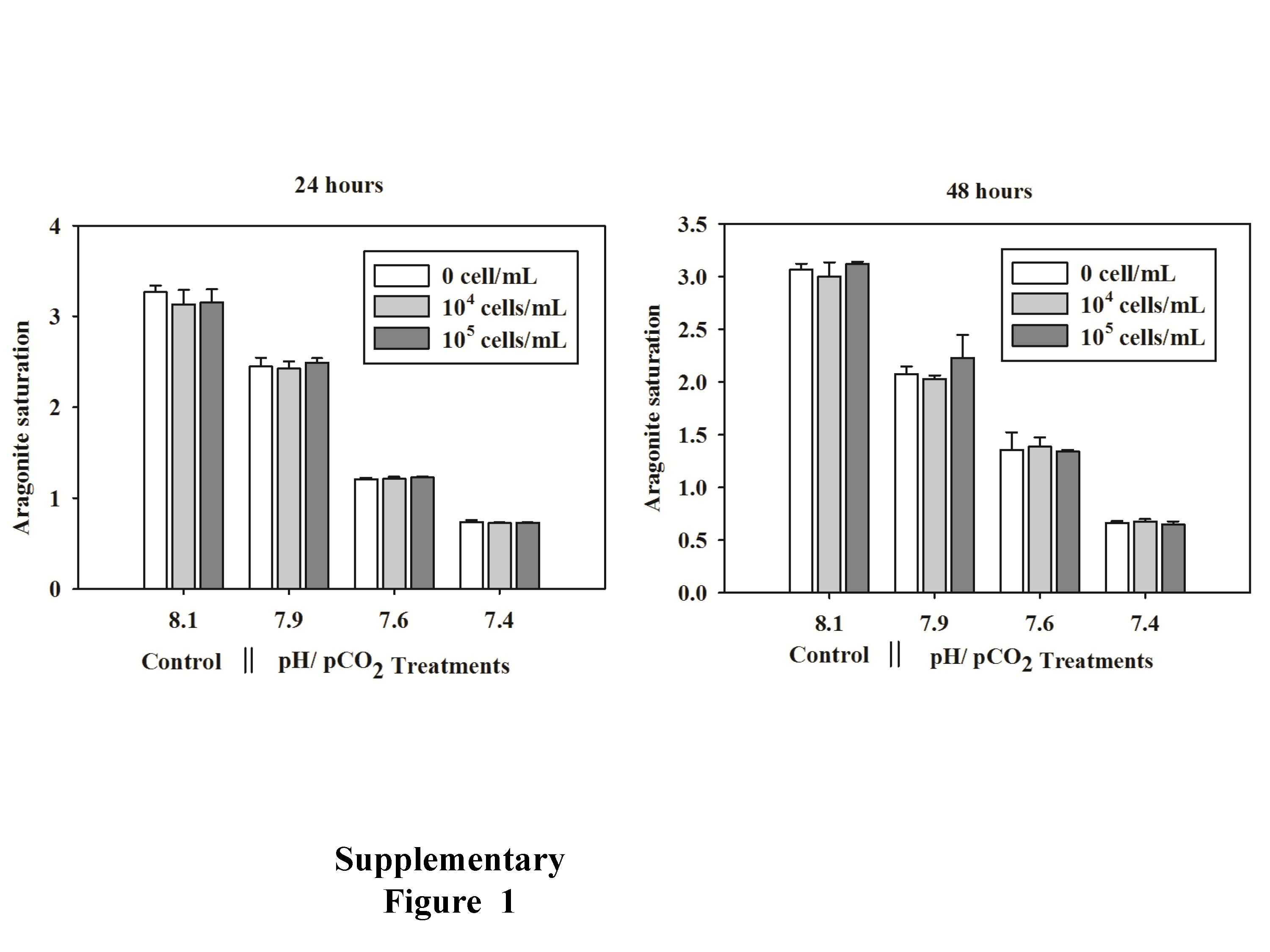

Supplement: Figure S1 — Effects of algae concentration on aragonite saturation state after 24 h and 48 h. This additional experiment without larvae examined the influence of three algal concentrations (0 cell/mL, 104 cells/mL, 105 cells/mL) on the aragonite saturation at four levels of pH (8.1, 7.9, 7.6, 7.4). Each bar represents the mean ± SD of 3 replicates. Three-way ANOVA: Algae; F2, 48 = 1.607; p>0.05; Algae*CO2; F6,48 = 1.444; p<0.05; Algae*Day; F3,48 = 0.183; p>0.05; Algae*CO2*Day; F6,48 = 0.640. (TIF) [file pone.0042718.s001.tif]
